# Student anxiety in introductory biology classrooms: Perceptions about active learning and persistence in the major

**DOI:** 10.1371/journal.pone.0182506

**Published:** 2017-08-03

**Authors:** Benjamin J. England, Jennifer R. Brigati, Elisabeth E. Schussler

**Affiliations:** 1 Division of Biology, The University of Tennessee, Knoxville, TN, United States of America; 2 Department of Biology, Maryville College, Maryville, TN, United States of America; 3 Department of Ecology and Evolutionary Biology, The University of Tennessee, Knoxville, TN, United States of America; National Brain Research Centre, INDIA

## Abstract

Many researchers have called for implementation of active learning practices in undergraduate science classrooms as one method to increase retention and persistence in STEM, yet there has been little research on the potential increases in student anxiety that may accompany these practices. This is of concern because excessive anxiety can decrease student performance. Levels and sources of student anxiety in three introductory biology lecture classes were investigated via an online survey and student interviews. The survey (n = 327) data revealed that 16% of students had moderately high classroom anxiety, which differed among the three classes. All five active learning classroom practices that were investigated caused student anxiety, with students voluntarily answering a question or being called on to answer a question causing higher anxiety than working in groups, completing worksheets, or answering clicker questions. Interviews revealed that student anxiety seemed to align with communication apprehension, social anxiety, and test anxiety. Additionally, students with higher general anxiety were more likely to self-report lower course grade and the intention to leave the major. These data suggest that a subset of students in introductory biology experience anxiety in response to active learning, and its potential impacts should be investigated.

## Introduction

The President’s Council of Advisors on Science and Technology [1] has projected a need for one million additional college graduates with STEM degrees in the next decade, a daunting goal given that fewer than 40% of freshmen STEM majors persist to earn a STEM degree. One of the recommendations of PCAST was the “widespread adoption of empirically validated teaching practices,” a call echoed by the American Association for the Advancement of Science’s meeting report *Vision and Change in Undergraduate Biology Education* [2]. Research indicates that the use of active learning in STEM courses, including group problem-solving, worksheets, tutorials, clickers, and peer instruction, leads to a lower failure rate [3, 4], increased understanding of course content [5, 6], and greater persistence in the major [3, 7, 8, 4].

In response, university instructors are incorporating more active learning techniques into their courses, with many institutions placing an emphasis on these teaching practices in large introductory STEM courses [9, 10, 11, 12] because of their higher attrition rates [13]. Recent research, however, has begun to investigate the variability in active learning outcomes and practices, with Eddy and Hogan [14] identifying that increased course structure disproportionately increased performance of black and first generation students, and Stains and Vickery [15] suggesting the need to assess variability in instructor active learning implementation. While studies often look at the effects of incorporating active learning into introductory courses through the lens of student performance, there has been relatively little research on how these classroom practices impact student anxiety. A recent review called student fear the “elephant in the classroom” [16]. Anxiety in response to active learning has been acknowledged via the suggestion to use implementation practices that reduce student apprehension [17]. However, studies directly measuring student anxiety are rare, with one investigating apprehension between genders for peer discussion [18], and another measuring anxiety in response to an active learning technique (cold calling) [19].

Student anxiety can be triggered by different types of classroom experiences. Evaluation anxiety, for example, is induced by the prospect of real or imagined social evaluation [20], and can include classroom communication apprehension (CCA), social anxiety, and test anxiety. Classroom communication apprehension (CCA) usually occurs because students are afraid they will appear inadequate in front of their peers or professors [21]. One study found that 70% of undergraduate students report feeling CCA at least occasionally [22]. Social anxiety, defined as a “marked and persistent fear of social or performance situations in which embarrassment may occur,” is estimated to impact 13% of people [23]. Test anxiety is also very common, with one study finding that 38.5% of undergraduates suffered from self-reported test anxiety [24]. Even low-stakes pop quizzes worth just 1% of a student’s grade can cause test anxiety [25].

While student reports of anxiety in the classroom are concerning, anxiety is not uniformly bad. Several studies have shown that students with at least some anxiety have higher test scores than their less-anxious peers [26, 27]. However, it is important to note that evaluation anxiety has been linked to avoidance of the feared situation and loss of motivation to perform, as well as decreases in working memory and attention [20]. Loss of motivation or self-efficacy as a result of anxiety could have important impacts on retention in the STEM major, given the relationship between these factors and resilience in student persistence and performance [28, 29].

This study emerged from a research project investigating instructor active learning practices and student perceptions of engagement in introductory biology classes in fall 2015. In open-ended survey responses, students spontaneously expressed anxiety about particular active learning practices being used by their instructors. Given these reports, data collection in spring 2016 was broadened to investigate general class anxiety and anxiety in response to specific classroom active learning practices. In this study, surveys and interviews of students in three large introductory biology courses investigated three hypotheses:

1. Some students in introductory biology will experience higher anxiety than others due to perceptions of evaluative anxiety in response to active learning.
2. Average student anxiety will vary depending on the active learning practices they experience and the lecture classes they attend.
3. Higher student anxiety will be associated with decreased student self-reported performance and persistence in the major.

## Materials and methods

### Courses and instructors

Survey and interview data were collected in spring 2016 from students attending one of three majors’ introductory biology lecture classes at a large Southeastern public research university. The introductory biology sequence at this university includes an Organismal and Ecological Biology (OEB) class (typically taken first) and a Cellular and Molecular Biology (CMB) class. Students from one section of OEB and two sections of CMB served as the potential participants for this study. Enrollment in each course ranged from 150–220 mostly freshman and sophomore biology or pre-professional majors. Classes met in large lecture format for two 50-minute class periods per week; the third 50-minute session each week was a GTA-led small group discussion focused on biological literacy. Students were only asked about their anxiety in the lecture portion of the class.

The instructors of the courses all held PhDs and had taught introductory biology courses previously. One CMB section (CMB 1) was co-taught by two instructors. One of the instructors taught the first half of CMB 1 and the second instructor taught the second half. All instructors were part of an introductory biology professional development community focused on active learning implementation in their courses.

The OEB instructor self-reported using clicker questions every class and worksheets regularly, but not every class. He asked questions to be answered by student volunteers or students he called on (cold calling) only occasionally. Students were often, but not always, allowed to discuss a question in groups before they were asked to respond. His clicker and worksheet questions were graded for both accuracy and participation. Up to 20% of the class grade depended on in class activities, and about half the activities were based on pre-class readings.

The CMB 1 instructor who taught for the first part of semester used clickers and worksheets almost every day. She asked students to answer questions often, but never cold called. Students could discuss clickers and worksheets in groups, but some work was individual. Worksheets were graded for participation and less than a third of the class activities were based on pre-class readings. The CMB 1 instructor who taught the second half of the course self-reported using clicker questions every class and worksheets often; she rarely asked verbal questions and never employed cold calling. Students were almost always allowed to discuss answers in groups. Clicker responses were graded for accuracy and participation, and worksheets were graded for accuracy. Most activities were based on pre-class readings. Less than 10% of the class grade was based on in-class activities.

The CMB 2 instructor self-reported using clicker questions and verbal questions with volunteer responses every class, and worksheet responses regularly, but not every class. She always allowed students to discuss questions prior to response. About 10% of her verbal questions also used cold calling. Her clicker questions and worksheets were for points, with some being graded for accuracy and some for participation. Up to 20% of the class grade depended on these classroom activities, and about half of the activities were based on pre-class readings.

All procedures for this study were approved by the University of Tennessee Institutional Review Board (IRB) for human subjects research. The university’s IRB ensures compliance with all federal and state regulations.

### Data collection

#### Survey

Data were collected through the use of an online survey (Qualtrics) sent via an e-mail link to students the final month of the semester by permission of the instructor. Instructors offered students one bonus point (out of 1,000 total course points) as an incentive for completing the survey.

One set of survey questions (N = 7 items) assessed students’ general class anxiety level on a Likert scale from 1–7, where 1 was no anxiety and 7 was high anxiety. These 7 items were from an existing sub-scale of a validated instrument designed to measure anxiety levels toward research [30]. For each of the seven items, the word research was replaced with Biology lecture. The prompt and each of the seven items were: Biology lecture…makes me nervous, is stressful, makes me anxious, scares me, is complex, is complicated, is difficult. These are reported in future sections as the “general class anxiety” data. A reliability analysis (Cronbach’s alpha) of the general class anxiety items indicated high reliability (α = 0.937).

We also wrote five survey items directly asking students to rank how anxious they became, on a scale from 1–5 (where 1 was not anxious at all and 5 was very anxious) when students: “respond to questions using clickers,” “are asked to volunteer to answer a question,” “are called on by name to answer a question,” “are asked to complete worksheets in class,” and “are asked to work in groups.” These practices were chosen because they had emerged in fall 2015 as practices students said caused them anxiety. These are reported in future sections as the “classroom practices anxiety” data.

In addition to their rankings of anxiety, student demographic information was collected regarding year in school, gender or gender identity, racial / ethnic identity, and current instructor. They were also asked their current grade in lecture class and whether they had changed their intended major since the beginning of the semester. The total time for survey completion averaged 5–10 minutes.

#### Interviews

Data were also collected via interviews of students from across the three lecture courses. Students were recruited through an early semester survey asking if they would be willing to be interviewed about their perspectives on classroom engagement and anxiety. Thirty three students said they were interested and were sent information about the study; 12 students then completed the scheduling process and were interviewed.

Due to scheduling constraints, the authors of this study each conducted four interviews. To assure consistency, each interviewer read a script for all questions, although there were opportunities to ask follow-up questions [31]. All interviews were conducted in a conference room adjoining the researchers’ lab space. Written consent was obtained prior to starting to the interviews, and verbal assent was obtained for audio recording. The interviews also included questions related to classroom engagement that were not included in this study.

During the interview, each student was provided a set of paper cards (Table 1) with typical classroom practices or occurrences and asked to select any that produced anxiety in their current biology class. The cards were created from factors previously found to impact student engagement or that fall 2015 students mentioned as causing them anxiety. The cards were always shuffled and laid out on the interview table in a random order as they were presented to students. After selecting the cards, students were asked to place them in order from most anxiety-producing to least anxiety-producing. Students could also add items that caused them anxiety on blank cards that were provided. For each card they chose, students were asked why it made them anxious and how it affected their engagement and/or learning. These card sorts and explanations are the basis for the interview results presented in this study.

**Table 1:** Classroom practice/ occurrence cards provided to students during interviews.

Answering clicker questions
Instructor asks a question and waits for a volunteer
Instructor asks a question and calls on a particular student
Completing in-class written activities or worksheets as an individual
Completing in-class written activities or worksheets with a group of your peers
Discussing a topic/problem with a group of your peers
Instructor provides a real-life example
PowerPoint includes visuals/graphics
A video is shown
Instructor uses humor / tells a joke
Instructor is enthusiastic
Instructor moves around the classroom
Instructor lectures
A topic you are interested in is being presented
Instructor does not explain the answer to a question
Instructor explains the answer to a question
Instructor explains a difficult concept

### Data analysis

#### Survey

All completed survey responses were downloaded to a database program (IBM SPSS version 22.0) for manipulation [32]. Any students who indicated that they were not over the age of 18 or who did not check the box giving permission for the use of their responses for the research project were removed from the data set.

For each student, his or her responses to the seven general class anxiety items were averaged to come to a single measure of general anxiety for each student, and then all student averages were compiled to generate summary statistics of mean, median, and variance for each of the three classes and for all classes together. To identify the proportion of students impacted by high general class anxiety across all classes, a distribution of the mean general class anxiety scores for all students was created. Cronbach’s alpha for the general class anxiety items was calculated using SPSS to assess internal consistency (reliability).

General class anxiety levels of students were compared based on their year in school using one-way ANOVA followed by Tukey’s post-hoc analysis. General class anxiety levels were compared between students of different genders and ethnicities (white vs. non-white) using two-tailed independent samples t-tests. Comparisons were considered significant at the p < 0.05 level.

Summary statistics (mean, median, variance) were calculated for student ratings of the anxiety they felt in response to each of the five classroom practices for all student respondents compiled. The mean student anxieties for each of the five classroom practices were then compared using one-way ANOVA followed by Tukey’s post-hoc analysis. For each classroom practice, differences between genders and ethnicities for students in all courses combined were compared using two-tailed independent samples t-tests.

The data were also analyzed to investigate potential differences in student anxiety among the three courses. General class anxiety differences among students in each of the three courses were compared using one-way ANOVA followed by Tukey’s post-hoc analysis. Student anxiety in response to each of the five classroom practices were analyzed for differences among the three courses using one-way ANOVA followed by Tukey’s post-hoc analysis. Effect sizes for the above analyses were calculated using Cohen’s f.

Anxiety levels were compared among students with different self-reported letter grades and intentions to stay or not stay in the biology major. Compiled student general class anxiety and anxiety in response to each of the five classroom practices were compared based on student self-reported letter grade (A, B, C, D, F) using one-way ANOVA followed by Tukey’s post-hoc analysis. For students who indicated that they were biology majors at the start of the semester (N = 129), student anxiety levels (general class anxiety and each of the five classroom practices) were compared between those who indicated that they were staying in the major versus switching out of the major using two-tailed independent samples t-tests. Effect sizes for the above analyses were calculated using Hedges’ g (t-tests) or Cohen’s f (ANOVA).

#### Interviews

The anxiety portions of each interview were transcribed fully and student explanations about why particular practices caused them anxiety underwent thematic analysis to identify consensus ideas expressed by students [33]. The first step of the analysis was to tally the cards chosen by each interviewee about practices that caused them anxiety. Practices (n = 7) chosen by more than three students were selected for further analysis. The explanations for why each of these practices caused student anxiety were identified from the interviews and compiled. One researcher read the responses about each practice and took notes on commonalities across the responses. Themes were created from these commonalities. A second researcher then was given the interview transcripts and themes and asked to check and confirm the themes. Afterwards, the researchers met to come to a consensus about the final themes. Once finalized, representative quotes were selected to illustrate each theme.

## Results

### Survey and interview participant demographics

The online survey had 327 usable responses (Table 2). At least 50% of each class population responded to the survey, but the responses were significantly skewed toward female responses versus male responses (course gender distribution was approximately even). A little over half of the respondents were freshman, with approximately a quarter being sophomores, and the rest were juniors or above. Almost 20% of the student respondents were non-Caucasian. There were no general class anxiety or classroom practice anxiety item differences based on gender, ethnicity or year in school for the participants overall.

**Table 2:** Demographics of students who completed the survey.

**Course**	**n**
OEB	102
CMB 1	70
CMB 2	154
**Year**	**n**
freshman	180
sophomore	94
junior	45
senior	7
super senior	4
**Gender**	**n**
female	215
male	112
**Race**	**n**
Caucasian	250
non-Caucasian	63

The interviewees consisted of nine females and three males; nine were Caucasians and three were non-Caucasians; five participants were freshmen, four were sophomores, one was a junior, and two were seniors. Four students were enrolled in OEB, one in CMB 1, six in CMB 2, and one student in both OEB and CMB 2 (Table 3). The twelve interviews averaged 33 minutes in length, with the shortest interview lasting 15 minutes and the longest lasting 95 minutes.

**Table 3:** Demographics of students who completed interviews.

**Course**	**n**
OEB	4
CMB 1	1
CMB 2OEB & CMB 2	61
**Year**	**n**
freshman	5
sophomore	4
junior	1
senior	2
super senior	0
**Gender**	**n**
female	9
male	3
**Race**	**n**
Caucasian	9
non-Caucasian	3

### Some students were more anxious than others, and some active learning practices caused more anxiety than others

**Fig 1:**
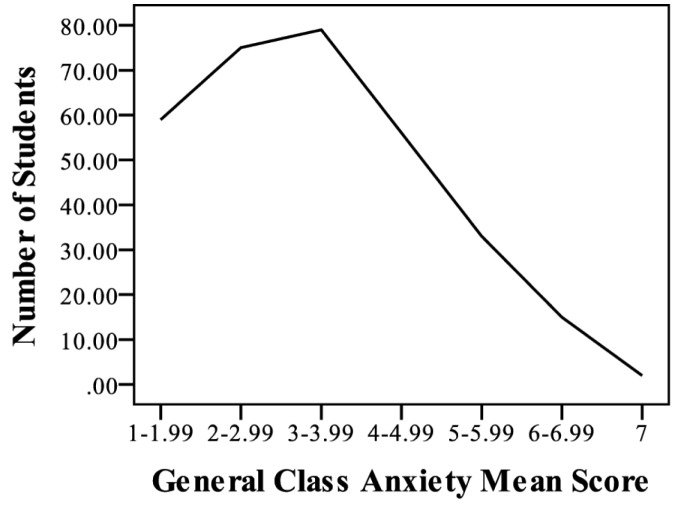
Distribution of general class anxiety means for all students in the study. Likert scale ranged from 1–7, with 1 being no anxiety and 7 being high anxiety. Line represents number of students per range of general anxiety means.

The distribution of general class anxiety student means is shown in Fig 1. The average general anxiety for all students was 3.29 ± 0.08 (standard error of the mean, SEM), which was slightly less than a mid-level anxiety. The distribution of general class anxiety means shows that 59 students had means between 1–1.99, 75 students had means from 2–2.99, 79 students had means from 3–3.99, 56 students had means from 4–4.99, 33 students had means from 5–5.99, 15 students had means from 6–6.99, and 2 students had means of 7. Thus, 16% of the total sample had higher than average general anxiety.

**Fig 2:**
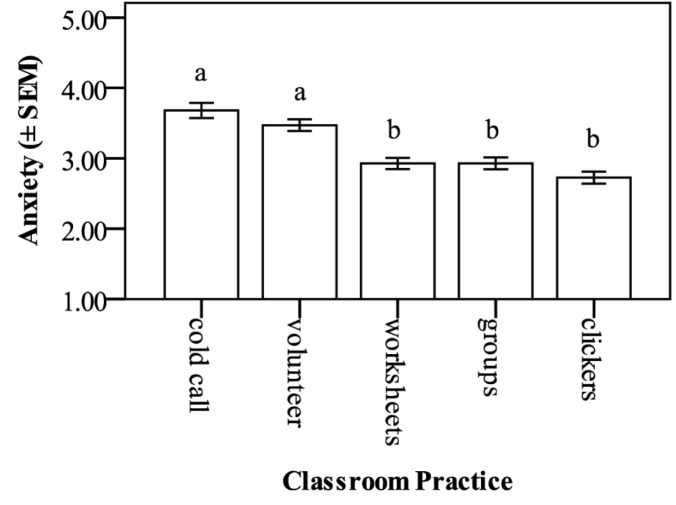
Average student anxiety for each of the five active learning practices. Cold calling caused significantly higher anxiety than completing worksheets, working in groups, or using clickers, but not higher than volunteering to answer (p < 0.001). Cohen’s f was 0.23, indicating a medium effect size. The Likert scale is 1–5, with 1 being not anxious at all, and 5 being very anxious. Data are mean anxiety ± standard error of the mean (SEM).

When comparing student anxiety in response to the five classroom practices (Fig 2), cold calling (3.68 ± 0.11) was found to have the highest student ratings of anxiety, followed by volunteering to answer a posed question (3.47 ± 0.08), completing worksheets (2.93 ± 0.08), working in groups (2.92 ± 0.08), and using clickers (2.73 ± 0.09). Each of the classroom practice anxiety means were above the mid-range of the Likert scale response range (1–5). Comparisons among the practices found that the overall significance of the model was p < 0.001, with cold calling and volunteering to answer a question not different from one another, but different from using clickers, working in groups, and completing worksheets. Effect size (Cohen’s f) was 0.23, indicating a medium effect.

### Anxiety levels varied by class/instructor

**Fig 3:**
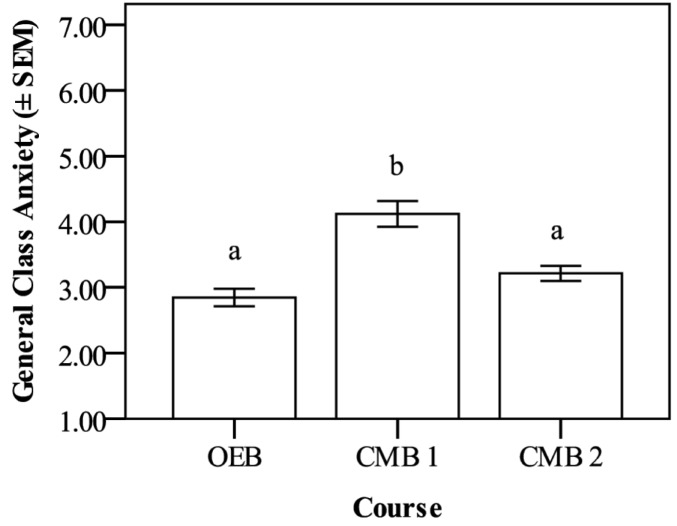
General class anxiety means showing significant differences among students in the three classes (p< 0.001). Students in CMB 1 had significantly higher general class anxiety than those in CMB 2 or OEB. Cohen’s f was calculated as 0.32, indicating a medium effect size. The Likert scale ranged from 1–7, with 1 being no anxiety and 7 being high anxiety. Data are mean anxiety ± standard error of the mean (SEM).

The mean general class anxiety for all three courses was 3.29 ± 0.08. Students in OEB had a mean general class anxiety level of 2.85 ± 0.14, those in CMB 1 had a mean of 4.12 ± 0.20, and those in CMB 2 had a mean of 3.21 ± 0.11 (Fig 3). CMB 1 student general class anxiety was significantly higher than for students in the other two classes, which were not different from each other (one-way ANOVA followed by Tukey’s post-hoc, p < 0.001). Effect size (Cohen’s f) was calculated as 0.32, indicating a medium-to-large effect.

**Fig 4:**
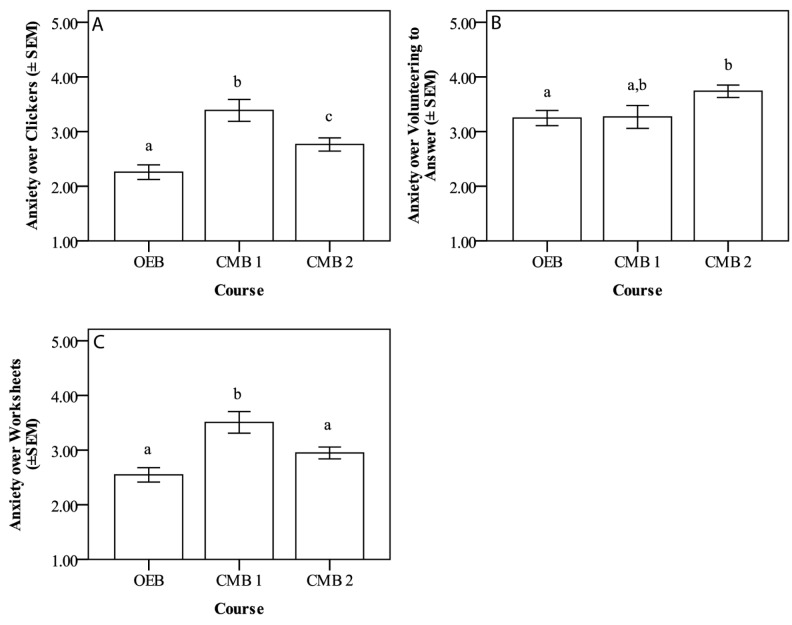
**Self-reported student anxiety toward using clickers (A), volunteering to answer a question in class (B), and completing worksheets (C) among the three lecture classes.** The Likert scale is 1–5, with 1 being not anxious at all, and 5 being very anxious. Data are mean anxiety ± standard error of the mean (SEM). (A) All three courses differ from one another in student-reported anxiety regarding the use of clickers (p < 0.001, Cohen’s f = 0.27, a medium effect size). (B) Students in OEB reported significantly lower anxiety than those in CMB 2 about volunteering to answer a question in class (p = 0.015, Cohen’s f = 0.17, a small effect size). (C) Students in CMB 1 reported higher anxiety about worksheets than students in OEB or CMB 2 reported (p < 0.001, Cohen’s f = 0.25, a medium effect size).

There were significant differences in student anxiety levels regarding the use of clickers among the three courses (Fig 4A). Students in OEB had a mean of 2.26 ± 0.13, those in CMB 1 had a mean of 3.39 ± 0.20, and those in CMB 2 had a mean of 2.76 ± 0.12, with Cohen’s f calculated as 0.27 (a medium effect). Significant differences were also found among classes for student anxiety levels when volunteering to answer a question, with students in OEB and CMB 2 being different from one another (Fig 4B). Students in OEB reported a mean of 3.24 ± 0.14, those in CMB 1 showed a mean of 3.27 ± 0.21, and those in CMB 2 showed a mean of 3.74 ± 0.11. Cohen’s f showed an effect size of 0.17 (a small effect). There were also significant differences regarding the use of worksheets, with students in CMB 1 (3.51 ± 0.20) reporting significantly higher levels of anxiety than students in CMB 2 (2.95 ± 0.11) or OEB (2.55 ± 0.13) (Fig 4C). Cohen’s f was calculated as 0.25, a medium effect.

### Students had different reasons for why different active learning practices caused anxiety

During interview card sorts, students indicated that a variety of active learning classroom practices caused them anxiety. The least commonly selected response among the card selections was “waiting on a volunteer” and “discussing a topic or problem with a group of peers,” which were each selected by three of the participants. All other cards were selected by at least 5 of the 12 participants, with the options of “working on in-class worksheets with peers” and “instructor calling on a student for an answer” being selected by the most students (7 and 8 students, respectively) (Table 4). One student selected no anxiety cards, and one student selected only one card. All other students selected at least 2 practices, with one student choosing 6 cards.

**Table 4:** Number of students (out of 12) who chose particular cards representing classroom practices that caused them anxiety, along with themes and representative quotes about why that practice caused them anxiety.

Anxiety-inducing option on card	Number who chose	Why it caused anxiety	Representative Quotes
Instructor asks a question and calls on a particular student	8	Putting you on the spot; not knowing the right answer	*“Sometimes I don’t know the answers and it makes me kind of anxious when they call on me and I am in front of the huge lecture hall and I don’t know what the answer is*.*”*
Completing in-class written activities or worksheets with a group of peers	7	Not knowing whether to trust the group’s work; finding others to work with	*“…because you’re with a group of people and you don’t really know them very well or there’s that one person who pipes up and talks a lot who thinks they know everything but you don’t really know if you can trust them or not*. *So I feel like with written activities in class it is sort of difficult because you were forced into a group you don’t really know and you don’t know if what you’re doing is heading in the right direction or in a negative direction or anything like that*.*”*
Completing in-class written activities / worksheets as an individual	6	Not sure if the answer is right and not having someone else to ask	*“Because I always second-guess myself if I’m not one-hundred percent sure of the answer*, *I don’t have anyone to ask for clarification… like hey… I think it’s this*, *am I on the right track or am I completely lost*?*”*
Answering clicker questions	6	Not getting the points	*“Specifically about timed clicker questions… you get like a minute and I end up stressing out*. *Sometimes I’ll think… I will have the right answer and then someone says something and I don’t have time to think about it and I change my answer*. *Time is awful*.*”* *“I can read all these pages but no one has explained it to me and I don’t completely understand them*, *so when I have to answer questions usually for points*, *if I don’t fully understand something then I’m going to miss out on points even though I’m in class and I’m trying*.*”*
Instructor does not explain answer to a question	5	Confusing	*“Because if we are have this question and we don’t get a clear answer for it then how are we supposed to study*?*”*
Instructor asks a question and waits for a volunteer	3	Takes too much time; awkward	*“I think it takes up a lot of time of class*. *There are some days when people are just not in the mood to answer questions and you just sit there and wait and you don’t know the answer so you don’t say anything and I think it just wastes a lot of time and ends up stressing me out because I want to get through the lecture and learn what we need to know*.*”*
Discussing a topic/problem with a group of your peers	3	Finding others to work with; making sure work is correct	*“I’m always afraid of saying something completely moronic in front of a peer*. *They might say you have no idea what you’re talking about do you*? *But the biggest part of that anxiety is trying to find someone when I’m sitting by myself*.*”*

When students were asked why each of the chosen practices caused them anxiety, students provided responses that indicated all three types of evaluative anxiety (see Table 4 for representative quotes). Group discussion and worksheets caused anxiety for some students because (1) some students had social anxiety (made them stressed to find others to work with), (2) students were afraid that they would misguide others, or that others would misguide them (and therefore they would lose points), and (3) some group members were not prepared and/or didn’t care about the assignment. Individual worksheets caused anxiety for some students because they were afraid they might not know the answers and wouldn’t be able to get help (and then they could lose points). Being called on to answer a question caused anxiety for some students because they (1) didn’t want to be put “on the spot,” and (2) had a fear of answering questions incorrectly in front of their instructor and/or a large group of their peers. The instructor asking a question and then waiting for a volunteer to answer caused anxiety for some students because the awkward silence made them uncomfortable and/or frustrated and they felt it wasted class time. Clicker questions caused anxiety for some students because they were worried about losing points. Often this anxiety arose because they felt it was unfair to be tested on something they had not discussed yet or because they didn’t have time to think about their response. The instructor not answering questions caused anxiety because it left them confused and unclear about what to study.

### High anxiety was related to lower self-reported grade and less intention to persist in the biology major

**Fig 5:**
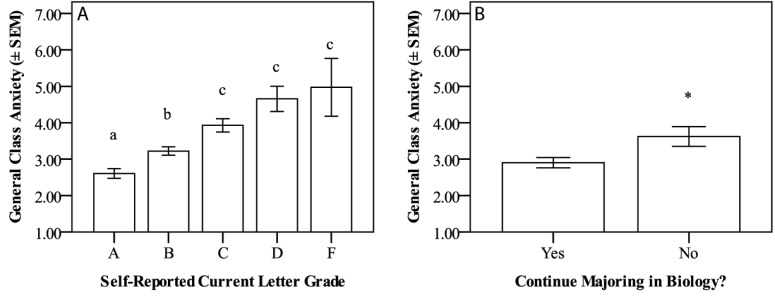
**General class anxiety compared among self-reported letter grade (A) and intention to persist in the biology major (B).** (A) mean general class anxiety scores on final survey based on self-reported letter grade in class. Students who indicated they were receiving a C, D, or F reported significantly higher anxiety than those who reported earning an A or B (p < 0.001). Cohen’s f = 0.43, indicating a large effect size. (B) Mean general class anxiety scores on final survey comparing students who indicated they were leaving the Biology major versus those who were continuing (p = 0.017). Hedges’ g = 0.50, indicating a medium effect size. Data are mean anxiety ± standard error of the mean (SEM).

Eighty-four students reported currently earning an A in their class; 145 reported a B; 69 reported a C; 12 reported a D; and 5 reported an F. Their mean general class anxiety sorted by self-reported course grade were: 2.61 ± 0.13 (self-reported an A); 3.22 ± 0.12 (self-reported a B); 3.93 ± 0.18 (self-reported a C); 4.65 ± 0.35 (self-reported a D); and 4.97 ± 0.79 (self-reported an F). Students who reported earning an A had significantly lower general anxiety than all other students. Those who reported earning a B had significantly higher general anxiety than those receiving an A, but less than that of those receiving a C, D, or F. Those who reported earning a C, D, or F had significantly higher general anxiety than those reporting earning an A or B (p < 0.001, Fig 5A). Cohen’s f was calculated as 0.43, a large effect. None of the five classroom practice anxiety measures were significantly associated with the students’ self-reported course grades.

Of the 327 students who completed the survey, 129 were Biology majors. One hundred of these students indicated that they were continuing in the biology major, but twenty-nine said they were not. There was a significant difference in mean general class anxiety between these two groups of students. Those who indicated they were not continuing in the major had significantly higher general class anxiety (p = 0.017, Fig 5B). The mean score for those leaving the major was 3.62 ± 0.27; the mean score for those remaining in the major was 2.90 ± 0.14. Effect size (Hedges’ g) was calculated as 0.50, indicating a medium effect. None of the five active learning practice anxiety measures were significantly associated with intention to stay in or leave the Biology major.

## Discussion

Although the use of active learning has been found to increase student learning in biology classes [4], this study found that active learning practices are also accompanied by perceptions of anxiety for some students taking introductory biology lecture classes. The classroom practices that students identified as causing them the highest anxiety overall were related to voluntarily answering or being asked to answer verbal questions the instructor posed, however, the level of anxiety for each active learning practice also depended on the class, suggesting fidelity of active learning implementation [15] may be a factor in anxiety. There were surprisingly no differences in student anxiety in relation to gender, ethnicity, or year in school for the overall sample, although our demographic inequality may have limited our ability to identify a significant effect. The sources of anxiety for each active learning practice were common across a small sample of interviewees and aligned with previous research on communication apprehension, social anxiety, and test anxiety [20]. There were associations between high general class anxiety and lower self-reported grades and not persisting in the Biology major; however, anxiety in response to active learning practices individually was not significantly related to self-reported success or persistence.

Of the types of active learning surveyed in these introductory courses, student anxiety about clicker use differed among all three courses, suggesting it may be the most sensitive to implementation differences. When speaking about clickers and anxiety, students mainly focused on the loss of points they caused, or the impact on their final grades. This supports recent research indicating that ungraded pop quizzes were more beneficial to students than graded quizzes because they do not cause student anxiety [25], however, it is also curious because students received points for participation as well as accuracy in all three classes. Students did not indicate communication or social anxiety in association with clicker use, even though all classes typically let students discuss or respond verbally to answers with other students before submitting a response. Although some might suggest that the technology or newness of clickers is the root cause of anxiety among students, these factors were not mentioned by students on the survey or in interviews.

Providing written responses to instructor questions also caused students to feel anxiety, but to a lesser extent than clickers and verbal responses. Anxiety was only higher for this practice in one of the classes, which was also the class where worksheets were used more intensively for the first part of the course. Interviews revealed that this practice caused all types of evaluative anxiety. Students mentioned, for example, that finding others to work with in large classes caused them stress (social anxiety), and that even when they found others to work with, they had to trust them to know the correct answers because the assignment was being turned in for a grade (test anxiety). This suggests that classrooms where group work is done for practice and not a grade may have lower levels of anxiety than those classrooms where written work is graded. In our classes, CMB 1 may have implemented worksheets more frequently and more often for a grade, making student anxiety higher for this practice. Even working on worksheets individually caused anxiety for some students because of the perception of the graded nature of the work. Students also mentioned that they were afraid to say something that would make them look stupid, thus suggesting that communication apprehension is also a factor. Clarifying for students why worksheets are being used and instructor expectations for group work may help students cope with this practice [34].

The last active learning practice that caused differential anxiety among the classes was volunteering to answer an instructor question. Although cold calling [19] was mentioned more often as an anxiety-inducing practice in the interviews and overall survey responses, its anxiety did not differ among the classes, likely because the instructors reported rarely using this practice. Interviewees mentioned that the source of anxiety was fear of looking stupid in front of their peers or classmates, a type of communication apprehension. Cold calling has been suggested as an instructional best practice because it helps promote equity in classrooms [17], but the practice also clearly causes anxiety disproportional to its use. Instructors would be well-served to talk with students about why they are using cold calling and to provide students with opportunities to discuss answers with classmates before calling on a student to buffer feelings of being singled out [21]. It may also be a good idea to use the practice on a daily basis to desensitize students to the anxiety, as suggested by McCroskey [35].

Of greatest concern in this study, and for many studying introductory biology more broadly, is that students who had higher general anxiety levels also self-reported lower grades in their course and a higher tendency to say they will leave the Biology major. Given that 16% of students across all three classes had above average anxiety (general anxiety mean ≥ 5), this suggests the need to study more closely what proportion of students in introductory biology are truly experiencing negative anxiety impacts on their persistence and success. One study found that students who reported high academic stress were 1.6 times more likely to consider leaving the STEM field [36]. There is no way in this study to assign causation or ascertain directionality, but it is likely that for a small percentage of students in these classes anxiety contributed to a negative course experience, and this is consistent with recent studies finding differential student outcomes with the use of active learning [14, 18]. However, one important caveat is that the associations between anxiety and self-reported success and persistence only existed for general class anxiety and not reports of anxiety for specific active learning practices. This suggests that anxiety that contributes to persistence and success is not necessarily about the individual active learning practices themselves, but about the complete active learning classroom experience (likely also including assessment).

This study suggests that more work needs to be done on the impacts of student anxiety as a result of active learning practices. We suggest that instructors become more aware of how their practices cause anxiety among their students, perhaps through surveys or feedback forms, and be more intentional about presenting students with their rationale for active learning use. This study did not examine interventions that might help anxious students, so studies should be conducted to identify and test the impact of coping interventions. We were also not able to determine how much of a factor course type or sequence had on the results. Finally, there is no way for us to know—from this study—the actual effects of anxiety on students’ learning; the anxiety may have been beneficial despite students’ self-reported grades [27].

### Limitations

This study was conducted by convenience sample, targeting three undergraduate introductory majors’ Biology courses. As the researchers could not control which students chose to participate, demographic parity among groups was unobtainable. The sample skewed heavily female, with almost twice as many females responding to the invitation as males. Additionally, response rates from each course varied. The disparate and overall smaller sample size did not allow for any sort of reliable logistic regression or discriminant function analysis, which would have allowed for a more thorough investigation of the relationship between anxiety, achievement, and persistence. It is also possible that the study’s sample size lacked the power to detect anxiety differences in gender, ethnicity, or year in school.

The researchers in this study used students’ self-reported letter grade at the end of the semester for the analysis. While this self-reported letter grade is important and may be indicative of how students perceive they are performing in the class, it is likely that the self-reported grades and actual earned final grades have a low probability of matching, as students often over- or under-estimate their chances of earning a higher grade at the end of the semester. Examining student anxiety based on actual earned letter grades may produce different results from what was obtained here. In the future, studies could collect both self-reported letter grade and actual earned final grade of student participants.

## Conclusions

Compared with traditional lecture, active learning instructional practices in large lecture classes introduce new challenges for students in the form of formative assessments and peer group work that were rarely a part of large classroom settings in the past. Although there were several psychological sources behind the anxiety students felt in this study, there were clearly also instructor effects that determined the level to which this anxiety manifested itself in the classroom. Although it is likely that many students were not negatively impacted by active learning anxiety given the strong evidence for its benefits [4], a certain percent of students reported high anxiety in these introductory courses that was statistically associated with lower self-reported course grade and persistence in the Biology major. Given the need to retain as many students as possible in science [1], there should be a more thorough investigation of the interplay between anxiety and active learning practices in large introductory science courses.

## Supporting information

S1 Dataset Raw data underlying the quantitative findings. (SAV)

S1 File Survey items and interview protocol. (DOCX)
