# Supplementary material for: Student anxiety in introductory biology classrooms: Perceptions about active learning and persistence in the major
Source: PLoS One. 2017 Aug 3;12(8):e0182506. doi: 10.1371/journal.pone.0182506 (PMC5542638; doi:10.1371/journal.pone.0182506)
Supplement: S1 File — (DOCX) [file pone.0182506.s002.docx]

**Online Survey (was disseminated electronically)**

This survey is asking about your level of anxiety* during Biology LECTURE class this semester.

*Anxiety and stress are different, but if you are feeling stress, you are probably also feeling anxiety.

If your instructor has decided to award you points for completion of this survey, you will receive those points at the end of the semester. Your instructor will not see the results of this survey until after grades have been turned in; all responses will be made anonymous before your instructor would view them. Therefore, the risk associated with your completing this survey is low.

This survey is being undertaken by the General Biology program to assess undergraduate learning in Biology at UT.

The survey is voluntary and asks you to answer a variety of questions, as well as provide demographic information. It may take 5-10 minutes to complete.

The IP address of your computer will not be recorded. However, all internet surveys have the potential for responses to be intercepted; therefore, we cannot guarantee confidentiality.

Your responses will not be "graded" on the basis of your responses. Any points given as an incentive will be based on completion of the survey and not particular responses.

Questions? Contact: Dr. Benjamin England, 974-3008, benglan6@utk.edu.

If you have questions about your rights as a participant, contact a Research Compliance Officer in the Office of Research at (865) 974-7697.

May we use your responses as part of our research related to Biology learning at UT?

- Yes
- No

Are you age 18 or above?

- Yes
- No

What year are you in school?

- 1 (freshman)
- 2
- 3
- 4
- > 4

What is your gender or gender identity?

- Female
- Male
- Prefer not to answer
- Open response______________

What is your racial/ethnic identity?

- Open response______________

What is the name of the professor of your Biology lecture class this semester?

- Professor 1
- Professor 2
- Professor 3

What do you think your current grade is in the lecture class?

- A
- B
- C
- D
- F

If you started this semester with the intention to major in Biology (or have already declared it as your major), are you still intending to major in it now?

- Yes
- No
- I did not start the semester intending to major in Biology, and still do not intend to major in Biology
- I did not start the semester intending to major in Biology, but am considering majoring in it now

Rate each of the following active learning practices based on how much ANXIETY they caused you to feel during class this semester. Use the following scale:

1 = This causes me no anxiety

3 = I feel moderately anxiety when this occurs in the class

5 = I feel very anxious when this occurs in class

| Students respond to questions using clickers 1 2 3 4 5 |
| --- |
| Students are asked to volunteer to answer a question 1 2 3 4 5 |
| A student is called on by name to answer a question 1 2 3 4 5 |
|  |
| Students are asked to complete worksheets in class 1 2 3 4 5 |
| Students are asked to work in groups 1 2 3 4 5 |

Please respond to the following items on a scale of strongly disagree to strongly agree.

|  |  |  | **Strongly disagree** | **Disagree** | **Somewhat disagree** | **Neither agree nor disagree** | **Somewhat agree** | **Agree** | **Strongly agree** |
| --- | --- | --- | --- | --- | --- | --- | --- | --- | --- |
| **Biology lecture makes me nervous** |  |  |  |  |  |  |  |  |  |
| **Biology lecture is stressful** |  |  |  |  |  |  |  |  |  |
| **Biology lecture makes me anxious** |  |  |  |  |  |  |  |  |  |
| **Biology lecture scares me** |  |  |  |  |  |  |  |  |  |
| **Biology lecture is complex** |  |  |  |  |  |  |  |  |  |
| **Biology lecture is complicated** |  |  |  |  |  |  |  |  |  |
| **Biology lecture is difficult** |  |  |  |  |  |  |  |  |  |

If your instructor is providing points, type your name here ___________________.

Thank you very much for completing this survey. Your responses have been recorded.

**Interview Protocol**

In this interview, we are going to be asking you about classroom teaching practices and anxiety in your biology class. Although anxiety and stress are different things, if you feel stress, it probably means you are experiencing anxiety.

All of the questions are based on your experiences in your biology class this semester.

Classroom teaching practices may include lecturing, using clickers, asking questions, or group discussions with classmates to answer a question or complete a worksheet.

There are no right or wrong answers to these questions, and these responses are completely confidential – we just want to gain your perspective about these ideas.

Since there are three people conducting interviews, I am going to be reading questions from a script.

1. What introductory biology courses have you taken at this university so far?

2. Who is your instructor this semester?

3. What is your major and what is your intended career? Has your intended career changed since arriving at this university?

4. Does class ever make you feel anxious? For this question, I will have you pick the cards that list activities that make you feel anxious, and then rank those cards from most anxiety-inducing to least. You may also create your own cards if you wish.

5. [for each card picked, ask students why that activity makes them anxious]
